# Supplementary material for: WordCommentsAnalyzer: A windows software tool for qualitative research
Source: F1000Res. 2018 Sep 4;7:536. Originally published 2018 May 3. [Version 2] doi: 10.12688/f1000research.14819.2 (PMC6137411; doi:10.12688/f1000research.14819.2)
Supplement: Supplementary file 3 [file f1000research-7-17614-s0002.tgz › b2a79496-4ada-47c4-9682-a99ce867836d.zip!/Eight Iranian Health Journals/Journal of Knowledge and Health_12.docx]

© Journal of Knowledge and Health. This work is licensed under CC BY 4.0 (https://creativecommons.org/licenses/by/4.0/).

Volume & Issue: دوره 10، شماره 2: 1394

Title: پيش‌بينی جهت و مقدار تغییرات فشار خون مردان میان سال غیرفعال به‌دنبال تمرین هوازی و بي‌تمريني

http://knh.shmu.ac.ir/index.php/site/article/view/545

مقدمه: به‌دليل احتمال بروز پاسخ‌های نامتعارف در فشارخون برخی افراد در سازگاری با تمرین و بي‌تمريني، ممکن است نسخه‌های ورزش توصیه شده توسط مراجع معتبر نیازمند بازنگری باشند. در این راستا، پيش‌بينی مقدار و نوع پاسخ‌دهي فشار خون افراد در سازگاری با تمرین و بي‌تمريني ضروری است. مواد و روش‌ها: تعداد 70 مرد میان سال غیرفعال داوطلب دارای فشارخون خفیف به‌طور تصادفی به دو گروه کنترل و تجربی تقسیم شدند. گروه تجربی به‌مدت هشت هفته در برنامه تمرین هوازی راه رفتن و دویدن (3 بار در هفته)، به‌مدت 40 دقیقه و با شدت 60 تا 70 درصد ضربان قلب بیشینه (MHR) شرکت کردند و در ادامه دو هفته بي‌تمريني وجود داشت. داده‌ها با استفاده از ضریب همبستگی پیرسون، آزمون خی دو، کاپا، تی همبسته و تحلیل واریانس اندازه‌گيري مکرر تحلیل داده‌ها انجام گرفت. نتایج: در هر دو مرحله تمرین و بي‌تمريني، نوع پاسخ‌دهي فشار میانگین سرخرگی نسبت‌به تمرین یکسان نبود، به‌طوری که برخی پاسخ‌دهي مناسب (GR)، برخی پاسخ‌دهي نامناسب (BR) و برخی دیگر عدم پاسخ‌دهي (NR) را تجربه کردند. مقادیر تمام شاخص‌های خطر متابولیک در طول تحقیق تغییر معني‌داری یافت، ولی تغییر امتیاز Z کلی خطر متابولیک (Zmets) فقط در آزمودنی‌های GR معني‌دار بود. آزمودنی‌های دارای پاسخ‌دهي مناسب نسبت‌به تمرین و همچنین آزمودنی‌های دارای پاسخ‌دهي نامناسب نسبت‌به بي‌تمريني، بیشترین تعداد شاخص‌های خطر متابولیک را در پیش آزمون دارا بودند. همچنين مقدار اولیه فشار میانگین سرخرگی، سن، شاخص توده بدن، LDL و HDL پلاسما به‌عنوان مهمترین پیش‌بینی‌کننده‌های تغییرات فشار میانگین سرخرگی در سازگاری با تمرین شناسایی گرديد. نتيجه‌گيري: در افراد دارای خطر متابولیک بالاتر، مشارکت در تمرین هوازی احتمالاً به افزایش فشارخون استراحتی منجر نمي‌شود. با این حال، به‌دليل کمبود شواهد تحقيقات بيشتري در اين زمينه پيشنهاد مي‌گردد.

Title: بررسی چگونگی ارتباط هوش معنوی و هوش هیجانی با سلامت روان در دانشجویان

http://knh.shmu.ac.ir/index.php/site/article/view/522

مقدمه: بهداشت روان از مهمترین موضوعات حوزه روانشناسی مي‌باشد. همچنین هوش معنوی و هیجانی با تقویت روابط فردی و معنویات می‌توانند باعث بهبود وضعیت سلامتی شوند. هدف این مطالعه بررسی ارتباط بین هوش معنوی و هوش هیجانی با سلامت روان مي‌باشد. مواد و روش‌ها: در این مطالعه مقطعی که بر روی 197 نفر از دانشجویان دانشگاه‌های استان سمنان در سال 1391 انجام شد. از پرسشنامه سلامت عمومی (GHQ-28) و هوش معنوی (Spirituality intelligence) و پرسشنامه هوش هیجانی (Emotional intelligence) بار- اون استفاده شده است. بررسی روابط بین متغیرها با استفاده از ضریب همبستگی پیرسون و آنالیز معادلات ساختاری (Structural equation model) انجام شده است. نتایج: بین نمره سلامت روان و نمره هوش معنوی همبستگی معکوس (32/0-r=) و بین نمره سلامت روان و نمره هوش هیجانی همبستگی منفی (26/0-r=) و همچنین بین نمره هوش معنوی و هوش هیجانی همبستگی مثبت (33/0=r) مشاهده شد. در پيش‌بينی سلامت عمومی از طریق هوش معنوی و هوش هیجانی مشاهده شد که هوش معنوی پيش‌بينی‌کننده معني‌داری برای سلامت روان مي‌باشد لیکن رابطه‌ی بین هوش هیجانی و سلامت روان معني دار نبود. نتيجه‌گيري: از آنجا که هوش معنوی بالاتر به سلامت روان بهتری مي‌انجامد لذا می‌توان با برنامه‌ریزی و آموزش‌های لازم در جهت بهبود کیفیت هوش معنوی دانشجویان اقدام نمود.

Title: اثرعصاره ريشه كودزو بر عوامل بیوشیمیایی سرم در موش‌هاي صحرايي ديابتي شده با استرپتوزوتوسين

http://knh.shmu.ac.ir/index.php/site/article/view/665

مقدمه: بیماری دیابت دارای عوارض مزمن جدی بوده و آنتی‌اکسیدان‌ها نقش مهمی در پاتولوژی، عوارض و کنترل این بیماری ایفا می‌نمایند. ریشه‌ی کودزو با قدمت زیاد در درمان سردردها، سرفه و ادم و به‌کار می‌رفته است. همچنین غنی از ایزو فلاونوئیدها بوده و دارای خواص آنتی‌اکسیدان می‌باشد. لذا در این مطالعه تأثير ريشه كودزو با‌ عنایت به خواص آنتي‌اكسيدان آن، بر برخی عوامل بیوشیمیایی سرم در موش‌هاي صحرايي ديابتي شده با استرپتوزوتوسين مورد آزمون قرار گرفت. مواد و روش‌ها: موش‌های نر نژاد ویستار به چهار گروه کنترل، دیابتی، دیابتی تیمار شده با mg/kg 100 عصاره ریشه کودزو و دیابتی تیمار شده با کودزو  mg/kg50 عصاره ریشه کودزو تقسیم شدند. دیابت به‌وسیله تزریق درون صفاقی STZ با دوز mg/kg 50 ایجاد گردید. تیمار با ریشه کودزو نیز با دوز mg/kg50 و 100 به‌مدت پنج هفته به‌صورت عصاره آبي و طي گاواژ انجام شد. همزمان عوامل بیوشیمیایی و هورمونی نیز مورد سنجش قرار گرفتند. نتايج: این مطالعه نشان داد، تزریق استرپتوزوتوسین موجب القای دیابت گردیده، متعاقب آن گلوكز، تري‌گليسريد و كلسترول تام افزایش و میزان HDL و انسولين سرم (01/0P≤) كاهش یافت. همچنین در نمونه‌هاي تيمارشده با ریشه کودزو ميزان گلوكز، تري‌گليسريد، كلسترول تام و LDL سرم كاهش يافته (01/0P≤) همچنین میزان HDL (01/0P≤) و انسولين سرم در هر دو گروه درمانی افزایش یافت. نتيجه‌گيري: این مطالعه نشان داد که ریشه کودزو علاوه بر خواص آنتی‌اکسیدان که در سایر مطالعات گذشته ذکر گردیده است مي‌تواند به‌عنوان مکمل دارویی در درمان عوارض ناشی از دیابت مورد استفاده قرار گیرد.

Title: اثر پیش ‌آماده‌سازی تمرین استقامتی بر مرگ سلولي و بیان پروتئین‌های Bax و Bcl-2 هیپوکمپ به‌دنبال ایسکمی- جريان مجدد مغزي در موش صحرایی نر

http://knh.shmu.ac.ir/index.php/site/article/view/672

مقدمه: ایسکمی- جريان مجدد مغزی موجب از دست دادن سلول‌های عصبی آسیب ديده از طریق مرگ برنامه‌ريزي شده در مناطق خاصی از مغز به‌ویژه هیپوکمپ مي‌شود. شواهد نشان مي‌دهد که ورزش آثار حفاظتي بر نورون‌ها دارد و ممکن است آسیب‌ نورونی ناشی از ایسکمی- جريان مجدد در موش‌ها را کاهش دهد. هدف از این مطالعه بررسی پیش‌آماده‌سازی فعالیت ورزشی بر بیان پروتئین‌های مرتبط با مرگ برنامه‌ريزي شده در نورون‌های ناحیه CA1 هیپوکامپ پس از القای ایسکمي ‌بود. مواد و روش‌ها: تعداد 21 سر موش صحرایی نر نژاد ویستار با وزن 300-260 گرم به‌طور تصادفی به سه گروه هفت تايي تقسیم شدند: گروه كنترل كاذب، گروه ايسكمي و گروه ورزش+ایسکمی. در گروه ایسکمی، حیوانات تحت ایسکمی تنها با استفاده از روش بستن دو شريان كاروتيد مشترك قرار گرفتند. در گروه ورزش+ایسکمی، حیوانات قبل از القاء ایسکمی، به‌مدت 4 هفته روی تردمیل دویدند وگروه كنترل كاذب که به‌عنوان شاهد در نظر گرفته شدند تحت عمل جراحی مشابه قرار گرفتند با این تفاوت که شریان کاروتید مشترک در آنها مسدود نمي‌شد. چهار روز بعد از ايسكمي سطوح بیان پروتئین‌هاي كاسپاز-3، Bax و Bcl-2 با تكنيك رنگ‌آمیزی ایمونوهیستوشیمی تعیین شد. نتايج: تعداد نورون‌های کاسپاز-3 مثبت در ناحيه CA1 گروه ایسکمی، در مقایسه با گروه شاهد، به‌طور معني‌داری افزایش یافت (001/0P<)، ورزش به‌طور قابل توجهی تعداد نورون‌های کاسپاز-3 مثبت ناشی از ایسکمی- جريان مجدد را در مقایسه با گروه ایسکمی کاهش داد (05/0P<). همچنین، نتایج افزایش قابل توجهی را نسبت Bax/Bcl-2 در گروه ایسکمی در مقایسه با گروه شاهد نشان داد (01/0P<). به‌علاوه، ورزش قبل از ایسکمی به‌طور قابل توجهی افزایش نسبت Bax/Bcl-2 ناشی از ایسکمی- جريان مجدد را کاهش داد (05/0P<). نتيجه‌گيري: این مطالعه نشان داده است ورزش، زمانی که به‌‌‌عنوان پیش‌آماده‌سازی استفاده مي‌شود، موجب آثار حفاظتی در برابر ایسکمی خواهد شد و ممکن است ورزش پيش استقامتي، یک استراتژی مفید برای کاهش عوارض مغزی ناشی از آسیب ایسکمیک مغزی باشد.

Title: تأثير ويتامين E بر استرس اكسيداتيو كبدي ناشي از ايسكمي- پرفيوژن مجدد كليوي در موش سوري

http://knh.shmu.ac.ir/index.php/site/article/view/675

مقدمه: مطالعات اخير نشان مي‌دهد كه آسيب ناشي از ايسكمي- پرفيوژن مجدد كليوي موجب ايجاد ضايعه در برخي اندام‌هاي دور دست مانند كبد مي‌گردد. از آنجايي كه برخي مطالعات، استرس اكسيداتيو را در ايجاد اين ضايعات دخيل مي‌دانند، در اين مطالعه اثر تجويز ويتامين E به‌عنوان يك آنتي‌اكسيدان بر استرس اكسيداتيو كبدي ناشي از ايسكمي- پرفيوژن مجدد كليوي مورد بررسي قرار گرفت. مواد و روش‌ها: موش‌هاي سوري نر به سه گروه نه تايي شم، گروه ايسكمي- پرفيوژن مجدد كليوي (45 دقيقه بستن دو طرفه شريان كليوي) و گروه ايسكمي- پرفيوژن مجدد كليوي + ويتامين  E(mg/kg 150، از طريق لوله دهاني به‌مدت 28 روز) تقسيم شدند. بعد از 24 ساعت پرفيوژن مجدد، حيوانات بيهوش شده و نمونه خون از قلب حيوان و همچنين بافت كبد جهت بررسي آنزيم‌هاي بافتي جمع‌آوري گرديد. نتايج: ايسكمي- پرفيوژن مجدد كليوي موجب افزايش غلظت پلاسمايي آلانين آمينوترانسفراز (AST) و آسپارت آمينو ترانسفراز (ALT) و كاهش فعاليت آنزيم‌هاي سوپر اكسيد ديسموتاز (SOD) و كاتالاز (CAT) در بافت كبد گرديد. مصرف ويتامين E موجب كاهش غلظت AST و ALT در پلاسما و افزايش غلظت SOD و CAT بافت كبدي نسبت‌به گروه ايسكمي- پرفيوژن مجدد كليوي گرديد. نتيجه‌گيري: يافته‌ها نشان مي‌دهد كه مصرف ويتامين E مي‌تواند موجب كاهش آسيب بافت كبد و حفظ آنزيم‌هاي آنتي‌اكسيدان كبد به‌دنبال استرس اكسيداتيو ناشي از IR كليوي مي‌گردد.

Title: مقایسه فعالیت ضد ویروسی عصاره‌های آبی و الکلی سیانوباکتری‌های جمع‌آوري شده از مناطق نفت‌خیز جنوب ایران

http://knh.shmu.ac.ir/index.php/site/article/view/607

مقدمه: سیانوباکتری‌ها دارای تنوع و گستردگی فراوانی هستند و خواص ضد باکتریایی آنها به اثبات رسیده است. هدف از مطالعه حاضر بررسی فعالیت و خواص ضد ویروسی سیانوباکتری‌های جمع‌آوري شده از مناطق آلوده به نفت جنوب ایران بود. مواد و روش‌ها: طی مطالعه حاضر عصاره آبی و متانولی پنج گونه از سیانوباکتری‌های جمع‌آوري شد، سپس تأثيرات سایتوتوکسیک آن بر روی رده‌های سلولی LCL و Hela، با استفاده از روش MTT در برابر کنترل منفی مورد ارزیابی قرار گرفت. جهت بررسی تأثيرات ضد ویروسی؛ سلول‌های LCL و Hela که به‌ترتيب آلوده به ویروس‌های ابشتن بار و پاپیلومای انسانی بودند با اين عصاره‌ها مواجهه یافتند. سپس استخراج DNA از نمونه‌ها صورت گرفت و PCR علیه ناحیه ژنی مسئول کد کردن پوشش ویروس انجام شد. همچنین آزمون توانایی تولید کلنی برای ویروس‌ها انجام شد. در پایان نتایج با استفاده از آزمون‌های آماری آنالیز واریانس دوطرفه با یکدیگر و با کنترل مقایسه شدند. نتایج: آثار سایتوتوکسیک بر روی سلول‌های LCL و Hela در گروه‌های تحت مواجهه با عصاره‌ها در مقایسه با یکدیگر و با گروه کنترل تفاوت معني‌داری داشت (01/0P<). همچنین نتايج بررسي ژني به ‌وسيله PCR کاهش معني‌دار Load ژنوم ویروس در گروه‌های تحت مواجهه با عصاره‌ها در مقایسه با کنترل و با یکدیگر را نشان داد. نتيجه‌گيري: باتوجه به آثار ضد ویروسی عصاره‌های الکلی و آبی سیانوباکتری‌ها، به‌نظر می‌رسد که مي‌توان از اين عصاره‌ها به‌عنوان کاندیداهای درمانی سود برد.

Title: تأثیر عصاره زعفران براختلالات یادگیری ناشی از ‌هایپوپرفیوژن مغزی

http://knh.shmu.ac.ir/index.php/site/article/view/562

مقدمه: گیاه زعفران گیاهی دارویی حاوی ترکیبات آنتی‌اکسیدان می‌باشد. مطالعه حاضر به‌علت استفاده از این خاصیت گیاه و همچنین نیاز جامعه به درمان و پیشگیری ضایعات شناختی که به‌دلیل افزایش استرس اکسیداتیو متعاقب ضایعات ایسکمیک مغز و بیماری‌های قلبی-عروقی‌ رخ می‌دهد، انجام شد. مواد و روش‌ها: در این مطالعه موش‌های صحرایی نر سفید به دو گروه آزمون و کنترل تقسیم شده و در گروه آزمون تجویز روزانهی عصارهی زعفران به‌مدت هفت روز صورت گرفت. گروه دیگر در همین مدت زمان سالین نرمال دریافت کرد. سپس با انجام عمل جراحی دو رگ (بستن عروق کاروتید مشترک) بر روی نیمی از نمونه‌های هر گروه، ضایعهکاهش جریان خون مغزی (هایپوپرفیوژن) در آنها ایجاد شد. سپس مجدداً به‌مدت 4 روزپیاپی بعد از عمل جراحی تمامی موش‌های گروه آزمون عصاره زعفران را دریافت نموده، در پایان همه موش‌ها با آزمون رفتاری ماز آبی موریس مورد ارزیابی قرار گرفتند. نتایج: آنالیز آزمون رفتاری ماز آبی موریس تفاوت معنی‌داری را میان گروه جراحی شده که دارو دریافت کرده بودند و گروه کنترل (جراحی شده بدون دریافت دارو) نشان داد. به عبارت دیگر عصاره زعفران موجب اختلالات یادگیری ناشی از هایپوپرفیوژن مغزی را به‌طور معنی‌داری کاهش داد. نتيجه‌گيري: نتایج این مطالعه حاکی از آن است که عصاره زعفران مي‌تواند اختلالات یادگیری ناشی از کاهش خو‌‌ن‌رسانی مغزی را که متعاقب ایجاد استرس اکسیداتیو و تخریب هیپوکمپ رخ مي‌دهد را در موش های صحرایی کاهش دهد.

Title: بررسی شأن ذاتی و عوامل مرتبط با آن در بیماران مبتلا‌به نارسایی قلبی

http://knh.shmu.ac.ir/index.php/site/article/view/353

مقدمه: احترام به شأن ذاتی انسان‌ها دارای نقش برجسته و اهمیت ویژه در پژوهش‌ها و بحث‌های مربوط‌به مراقبت و سلامت مي‌باشد. پژوهش حاضر با هدف تعیین شأن ذاتی و عوامل مرتبط با آن در بیماران مبتلا‌به نارسایی قلبی صورت گرفته است. مواد و روش‌ها: يك مطالعه توصيفي- مقطعی بوده و جامعه مورد مطالعه آن بیماران مبتلا‌به نارسایی قلبی بستری در بخش قلب بيمارستان‌هاي حضرت رسول (ص)، امام حسین (ع) و شریعتی تهران بودند. در اين پژوهش، نمونه‏گيري به‌صورت مبتنی بر هدف انجام گرفته و تعداد 130 بيمار مبتلا‌به نارسایی قلبی از ميان جامعه پژوهش انتخاب گرديدند سپس پرسشنامه ويژ‌گي‌هاي فردي و پرسشنامه شأن ذاتي (IDQ)، توسط آنان تكميل گرديد. تجزیه ‌و‌ تحلیل آماری با استفاده از روش‌هاي آماري توصيفي و آزمون‌های آماری پیرسون، آنالیز واریانس یک طرفه و تی مستقل انجام شد. نتایج: میانگین نمره شأن ذاتی در واحدهای مورد پژوهش 8/0 ± 6/4 محاسبه گردید. بیشترین میانگین نمره مربوط‌به بعد شأن ذاتی در خانواده و کمترین میانگین نمره مربوط‌به بعد شأن ذاتی در جامعه مورد پژوهش به‌دست آمد همچنين آزمون آماری آناليز واريانس، تفاوت معني‌داري را بین میانگین نمره شأن ذاتی در دو بعد ذکر شده نشان داد. آزمون آماری پيرسون، نيز ارتباط معني‌دار آماری بین سن با نمره شأن ذاتي و بین شدت بیماری (كسر تخليه‌اي بطن چپ) با نمره شأن ذاتي نشان داد. نتيجه‌گيري: باتوجه به اهمیت نقش یکایک افراد در خانواده، جامعه و سیستم‌های بهداشتی- درمانی و تعهد آنان در حفظ و ارتقاء شأن بیماران، نتایج پژوهش حاضر مي‌تواند در برنامه‌ریزی جهت حمایت و بهبود درمان و مراقبت از بیماران و هدایت پژوهش‌های بعدی مرتبط با شأن ذاتی در بیماران مختلف سودمند باشد.

Title: نقش مداخلات تغذیه‌اي در پیشگیری و کنترل دیابت بارداری: یک مرور نظام‌مند

http://knh.shmu.ac.ir/index.php/site/article/view/551

مقدمه: دیابت بارداری نوعی از دیابت است که اولین بار در طی حاملگی تشخیص داده مي‌شود و شیوع آن در ایران بین 3/1% تا 9/8% گزارش شده است. این مطالعه با هدف مقایسه مداخلات تغذیه‌اي بدون آموزش، مبتنی بر روش‌های آموزشی و آموزش با استفاده از مدل‌های تغییر رفتار برای کنترل دیابت بارداری انجام شد. مواد و روش‌ها: جستجوی الکترونیکی با استفاده از کلمات کلیدی فارسی و انگلیسی از پایگاه‌های بانک اطلاعاتی مقالات علوم پزشکی ایران (Iran Medex)، پایگاه اطلاعات علمی (SID)، ScienceDirect، PubMed/MEDLINE انجام شد. مطالعات انتخاب شده به 3 دسته مقایسه رژیم‌های غذایی، آموزش رژیم غذایی مبتنی‌بر تئوری‌ها و مدل‌های تغییر رفتار آموزش رژیم عذایی بدون مدل تقسیم گرديد. نتايج: در مجموع رژیم پر فیبر در کنترل قند خون در همه مطالعات مؤثر بوده و حمایت ا جتماعی، خودکارآمدی و دریافت بازخود در اتخاذ رفتارهای سالم نيز تأثير به‌سزايي داشت، همچنين آموزش گروهی نسبت‌به آموزش‌های چهره به چهره مؤثرتر بودند. نتيجه‌گيري: نتايج اين پژوهش نشان داد كه سازمان‌دهي و برنامه‌ريزي آموزش‌هاي مناسب و متناسب با نيازهای مخاطبان با استفاده از مدل‌ها و تئوری‌های آموزش بهداشت به جای آموزش‌های رايج امري ضروری به‌نظر مي‌رسد.

Title: بررسی ژنتیکی و پروتئینی بتا دفنسین 126 و ارتباط آن با نتایج درمان ناباروری از طریق تزریق داخل رحمی اسپرم

http://knh.shmu.ac.ir/index.php/site/article/view/595

مقدمه: بتا دفنسین 126 (DEFB 126) انسانی یک گلیکوپروتئین کوچک کاتیونی است که به‌عنوان جزء مهمی از گلیکوکالیکس اسپرم پریمات‌ها و انسان مطرح مي‌شود. این پروتئین از اسپرم در مقابل میکروب‌های عفونت‌زا و سیستم ایمنی زنان محافظت مي‌کند. ژن DEFB126 در انسان روی انتهای تلومری بازوی کوتاه کروموزوم 20 قرار دارد. یک تغییر ژنتیکی در چارچوب خوانش این ژن که در اثر حذف دو سیتوزین به‌وجود مي‌آید، عملکرد اسپرم را تحت تأثير قرار مي‌دهد. لذا این مطالعه با هدف تعیین ارتباط این جهش با میزان موفقیت در تزریق داخل رحمی اسپرم (IUI) انجام گرفت. مواد و روش‌ها: در اين مطالعه میزان جهش ژن DEFB126 در 76 مرد ایرانی با دلایل نامشخص ناباروری که زنان آنها نتایج مثبت یا منفی IUI داشتند مشخص گرديد. پس از استخراج DNA از خون بیماران مورد مطالعه، کیفیت DNA استخراج شده توسط دستگاه اسپکتروفوتومتر مورد ارزیابی قرار گرفت. روش‌های (PCR-SSCP) و Sequencing نيز برای تأيید نتایج استفاده شدند. علاوه بر این از تكنيك  ایمونوسیتوشیمی برای بررسی میزان بيان این پروتئین در سطح سلول‌های اسپرم استفاده گرديد. نتايج: نتایج بررسي DNA نشان داد که 4/24 درصد از مردانی که نتایج باروری همسرانشان منفی شده بود دارای جهش مي‌باشند، در‌حالی که افرادی که نتایج باروری آنها مثبت شده بود فاقد این جهش بودند. همچنین پروتئین کمتری بر سطح اسپرم مردان دارای آلل جهش‌دار مشاهده شد. نتيجه‌گيري: نتايج مطالعه حاضر نشان مي‌دهد که داشتن آلل هموزیگوت جهش‌دار در توالی بتا دفنسین 126 عامل مؤثری در عدم موفقیت درمان از طریق IUI مي‌باشد.
